# CAZymes from the thermophilic fungus *Thermoascus aurantiacus* are induced by C5 and C6 sugars

**DOI:** 10.1186/s13068-021-02018-5

**Published:** 2021-08-12

**Authors:** Raphael Gabriel, Rebecca Mueller, Lena Floerl, Cynthia Hopson, Simon Harth, Timo Schuerg, Andre Fleissner, Steven W. Singer

**Affiliations:** 1 Biological Systems and Engineering Division, Lawrence Berkeley National Laboratory, Berkeley, CA 9720 USA; 2 Joint BioEnergy Institute, Emeryville, CA 94608 USA; 3 Institut Für Genetik, Technische Universität Braunschweig, Spielmannstr. 7, 38106 Braunschweig, Germany; 4 Department of Applied Genetics and Cell Biology, University of Natural Resources and Life Sciences Vienna (BOKU), Muthgasse 18, 1190 Vienna, Austria; 5 Laboratory of Food Systems Biotechnology, Institute of Food, Nutrition and Health, ETH Zurich, Zurich, Switzerland; 6 Department of Chemical Engineering and Materials, Faculty of Chemistry, Complutense University of Madrid, Av. Complutense s/n, 28040 Madrid, Spain; 7 Frankfurt Institute of Molecular Biosciences, Goethe University Frankfurt, 60438 Frankfurt am Main, Germany; 8 Braunschweig Integrated Centre of Systems Biology (BRICS), Rebenring 56, 38106 Braunschweig, Germany

**Keywords:** CAZy, Filamentous fungi, *Thermoascus aurantiacus*, Transcriptomics, Cellulase gene expression

## Abstract

**Background:**

Filamentous fungi are excellent lignocellulose degraders, which they achieve through producing carbohydrate active enzymes (CAZymes). CAZyme production is highly orchestrated and gene expression analysis has greatly expanded understanding of this important biotechnological process. The thermophilic fungus *Thermoascus aurantiacus* secretes highly active thermostable enzymes that enable saccharifications at higher temperatures; however, the genome-wide measurements of gene expression in response to CAZyme induction are not understood.

**Results:**

A fed-batch system with plant biomass-derived sugars d-xylose, l-arabinose and cellobiose established that these sugars induce CAZyme expression in *T. aurantiacus*. The C5 sugars induced both cellulases and hemicellulases, while cellobiose specifically induced cellulases. A minimal medium formulation was developed to enable gene expression studies of *T. aurantiacus* with these inducers. It was found that d-xylose and l-arabinose strongly induced a wide variety of CAZymes, auxiliary activity (AA) enzymes and carbohydrate esterases (CEs), while cellobiose facilitated lower expression of mostly cellulase genes. Furthermore, putative orthologues of different unfolded protein response genes were up-regulated during the C5 sugar feeding together with genes in the C5 sugar assimilation pathways.

**Conclusion:**

This work has identified two additional CAZyme inducers for *T. aurantiacus*, l-arabinose and cellobiose, along with d-xylose. A combination of biochemical assays and RNA-seq measurements established that C5 sugars induce a suite of cellulases and hemicellulases, providing paths to produce broad spectrum thermotolerant enzymatic mixtures.

**Supplementary Information:**

The online version contains supplementary material available at 10.1186/s13068-021-02018-5.

## Introduction

Carbohydrate active enzymes (CAZymes) are vital for the conversion of plant polysaccharides to biofuels and bio-based chemicals [1]. Cellulose and hemicellulose are the most abundant polysaccharides in plant biomass and thus are an immense untapped carbon pool for biotechnological applications. Through using CAZymes such as cellulases and xylanases, lignocellulosic plant biomass can be deconstructed into simple sugars that can be further converted into biofuels and bioproducts [2].

The thermophilic fungus *Thermoascus aurantiacus* is a notable host for thermostable CAZyme production [3]. The enzymes of this fungus were found to be more heat stable and effective at deconstructing lignocellulose than enzymes from other thermophilic fungi and demonstrated the release of sugars from pre-treated biomass at comparable levels to the commercial enzymatic mixture CTec2 at 50 °C. Notably, the enzymatic mixture from *T. aurantiacus* lost only half of its activity during saccharification of pre-treated switchgrass at 70 °C, while CTec2 was inactive at this temperature [4]. Moreover, *T. aurantiacus* secretes large amounts of a lytic polysaccharide monooxygenase (LPMO), which depolymerizes cellulose and has been extensively studied and shown to be the most abundant protein secreted by *T. aurantiacus* [5]. Recently, procedures for genetic transformation, sexual crossing and gene editing using CRISPR/Cas9 have been established for this self-crossing organism, which opens many possibilities to harness the ability of *T. aurantiacus* to secrete highly active and thermostable CAZymes [6, 7]. However, little is known about CAZyme induction in *T. aurantiacus*, which is vital for designing improved strain engineering and bioprocess strategies.

Cellulase and xylanase induction has been intensively studied in a few filamentous fungi [8–11]. *Trichoderma reesei* has historically been the most prominent fungus for commercial cellulase production [12, 13]. The cellulases of *T. reesei* are induced in the presence cellulose as well as simple sugars like cellobiose, lactose and sophorose. While cellobiose induces cellulases in a variety of filamentous fungi, including the model fungus *Neurospora crassa*, cellulase induction by lactose and sophorose is limited to a few fungi besides *T. reesei* [14]. For *N. crassa* and *T. reesei*, xylose induces xylanase production; however, xylose was found to induce both cellulases and xylanases in the industrially used host *Aspergillus niger* [15]. While less is known about the inducing effect of l-arabinose, another major plant cell wall sugar, it was reported that this sugar induced xylanases in *N. crassa* and *T. reesei* [16–18]. These findings indicate that sugars related to plant cell wall degradation have very different effects on CAZyme secretion, even in closely related fungal species.

Induction of CAZymes by *T. aurantiacus* is not well understood. It was initially reported that *T. aurantiacus* secreted the highest level of cellulases when grown on alkali pre-treated bagasse and xylan while weak cellulase activity was found on Avicel cellulose [8]. To identify further inducers for *T. aurantiacus*, batch cultivations with d-xylose, cellobiose and l-arabinose were performed. High xylanase secretion of *T. aurantiacus* was reported for batch-cultures of xylan and non-metabolizable methyl β-d-xylopyranoside (MXP), but not xylobiose and d-xylose [10, 19]. Recently, experiments using controlled feeding of d-xylose to *T. aurantiacus* cultured in shake flasks and bioreactors demonstrated substantial cellulase and xylanase induction that was higher than batch cultures with xylan [11]. Studies performing similar fed-batch experiments using *T. reesei* found strong CAZyme secretion induction with sugars related to plant cell wall degradation [17, 20]. Therefore, continuous feeding helps to circumvent two challenges associated with batch cultures: (1) the high initial amount of sugar first causes carbon catabolite repression (CCR) [21]; the sugar is then consumed rapidly, leading to no further induction; and (2) the presence of impurities in highly purified plant polysaccharides (e.g., cellulose contaminated with residual xylan) that can lead to fundamentally different outcomes compared to using purified sugar substrates at low concentration [11]. Lastly, fed-batch studies can be of great utility for -omics experiments to refine bioprocess and genetic engineering strategies for generating high enzyme secreting fungal strains [22–24].

The goal of this study was to test CAZyme inducers of *T. aurantiacus* by fed-batch cultivation to screen for enzymatic activities and to reveal how gene expression is affected by these inducers.

## Results

### Arabinose and cellobiose induce CAZymes in *T. aurantiacus*

**Fig. 1:**
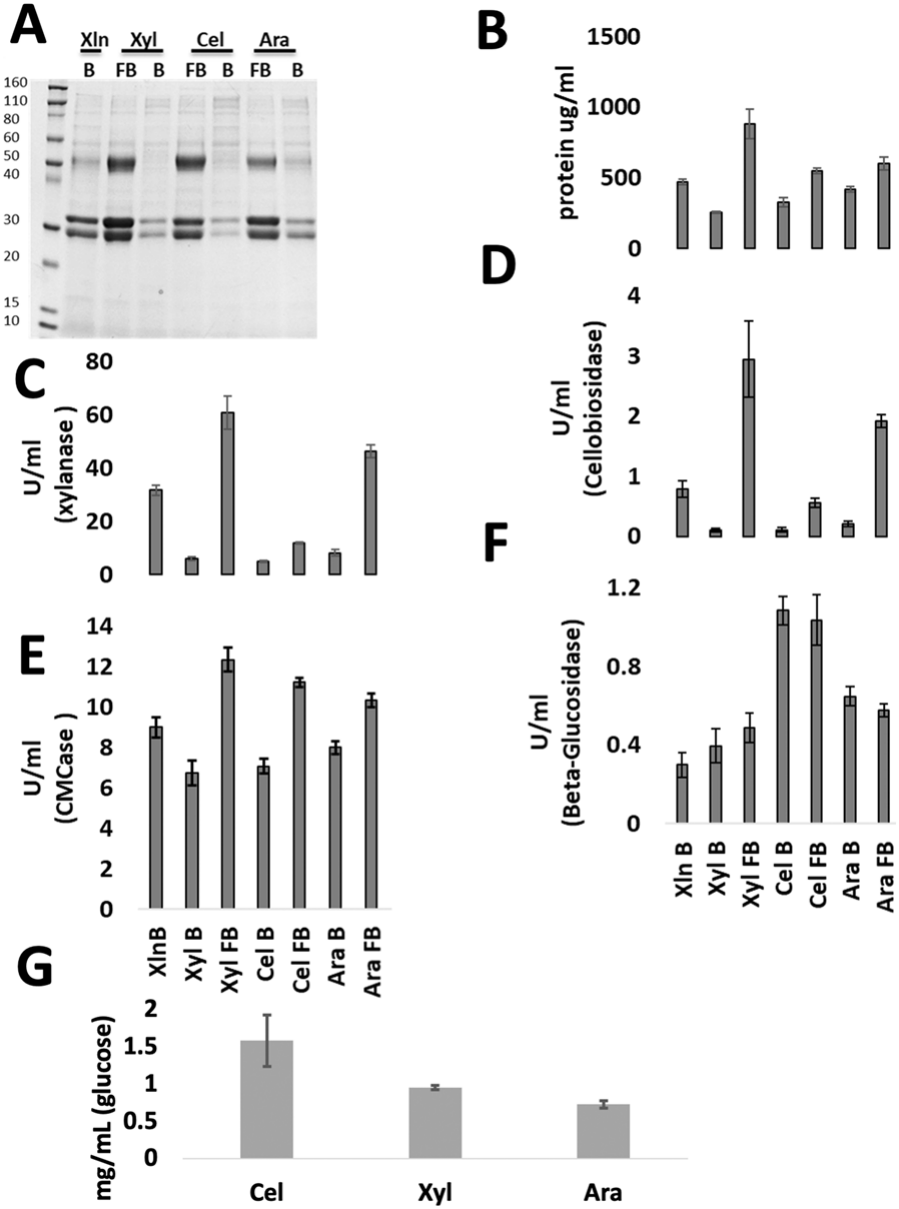
Comparison of CAZyme secretion of *T. aurantiacus* in fed-batch (FB) and batch (B) cultures with beechwood xylan (Xln), d-xylose (Xyl), cellobiose (Cel) and l-arabinose (Ara) and through **A** SDS PAGE with molecular weight marker in kDa with 15 µl filtered culture supernatant loaded per lane (identities of the prominent bands are noted in the Results); **B** protein amount with the Bradford Protein Assay; **C** DNS-xylanase assay; **D** pNPC-cellobiosidase assay; **E** DNS-CMCase assay and **F** pNPG-β glucosidase assay; **G** Glucose release from Avicel using filtrates from FB cultures (error bars represent standard deviations, *n* = 3)

Previously, fed-batch induction experiments led to the identification of d-xylose as an inducer of CAZymes in *T. aurantiacus* [11]. During this previous fed-batch study, beechwood xylan and a variety of celluloses (Avicel, Sigmacell, bacterial cellulose) also induced CAZyme production in *T. aurantiacus*. Since both beechwood xylan and the plant-derived celluloses (Avicel, Sigmacell) contained xylose, the inductive effects of other sugars besides xylose in these substrates could not be deconvoluted [24]. Therefore, we chose two additional lignocellulose-derived sugars to test as inducers: l-arabinose, which constitutes ~ 10% of beechwood xylan, and cellobiose, which is a common cellulase inducer and the product of cellobiohydrolase [11]. *T. aurantiacus* was grown first in a seed culture in d-glucose medium supplemented with soy meal peptone for 48 h and shifted to two sets of shake flasks; one set containing each individual sugar (0.5% w/v xylose, cellobiose and l-arabinose, referred to as batch culture set) and another set of flasks where the same amount of those sugars were continuously fed (fed-batch set) over a 3-day period (Fig. 1). The batch culture set also included triplicate beechwood xylan (0.5% w/v) cultures to compare the purified sugars with a complex substrate that previously found to be a good CAZyme inducer.

SDS-PAGE of culture filtrates demonstrated that the characteristic CAZyme bands previously observed for *T. aurantiacus* (cellobiohydrolase (~ 54 kDa), endoglucanase/xylanase (~ 33 kDa), and lytic polysaccharide monooxygenase (~ 25 kDa) [3, 4] were clearly visible for the fed-batch cultures, which had higher total protein titers than batch cultures (Fig. 1A and B). The three sugar feeds induced different enzymatic activities. High xylanase activities were found during C5 sugar feeding, while cellobiose fed-batch led to a small increase in xylanase activity in the culture broth (Fig. 1C). Interestingly, cellobiosidase activity followed the same trend of high activity during C5 sugar feeding (Fig. 1D) while endoglucanase activities, measured through the CMCase assay, were in a comparable range for both the cellobiose and C5 fed-batch cultures (Fig. 1E). In contrast, the cellobiose fed-batch and batch cultured displayed higher β-glucosidase activity than the C5 sugar cultures (Fig. 1F). The ability of the supernatants from the fed-batch cultures to release glucose from Avicel was also tested. In these experiments, the supernatant from the cellobiose-grown cultures had 72% higher glucose release than the xylose-grown cultures and 114% higher glucose release than the arabinose-grown cultures (Fig. 1G).

### Development of minimal medium for *T. aurantiacus* protein production

The fed-batch experiments identified d-xylose, l-arabinose and cellobiose as inducers of CAZyme production. Gene expression experiments were designed to study gene expression patterns under these growth conditions to describe the cellular responses to these sugars. However, the *T. aurantiacus* induction experiments described above were performed with soy meal peptone, a complex nitrogen source containing sugars. Peptones can pose challenges when using systems biology tools, since considerable differences among manufacturers and also batch effects for the same product can be found [25]. While peptones were found to enable high protein production of *T. aurantiacus*, using a defined medium appeared more favorable for gene expression experiments. Therefore, a minimal medium with a defined nitrogen source was designed in this study. Previously, *T. aurantiacus* cultivations have been performed on media containing complex nitrogen sources such as yeast extract or peptones [4, 8, 9, 19, 26–31]. In only one study, *T. aurantiacus* was cultivated in Vogel’s minimal glucose medium [10]. This medium supported poor growth in our hands, so the McClendon’s medium, which has been used for the above described CAZyme production was adapted to employ (NH_4_)_2_SO_4_ as the sole nitrogen source for a defined minimal medium to study *T. aurantiacus* induction.

**Fig. 2:**
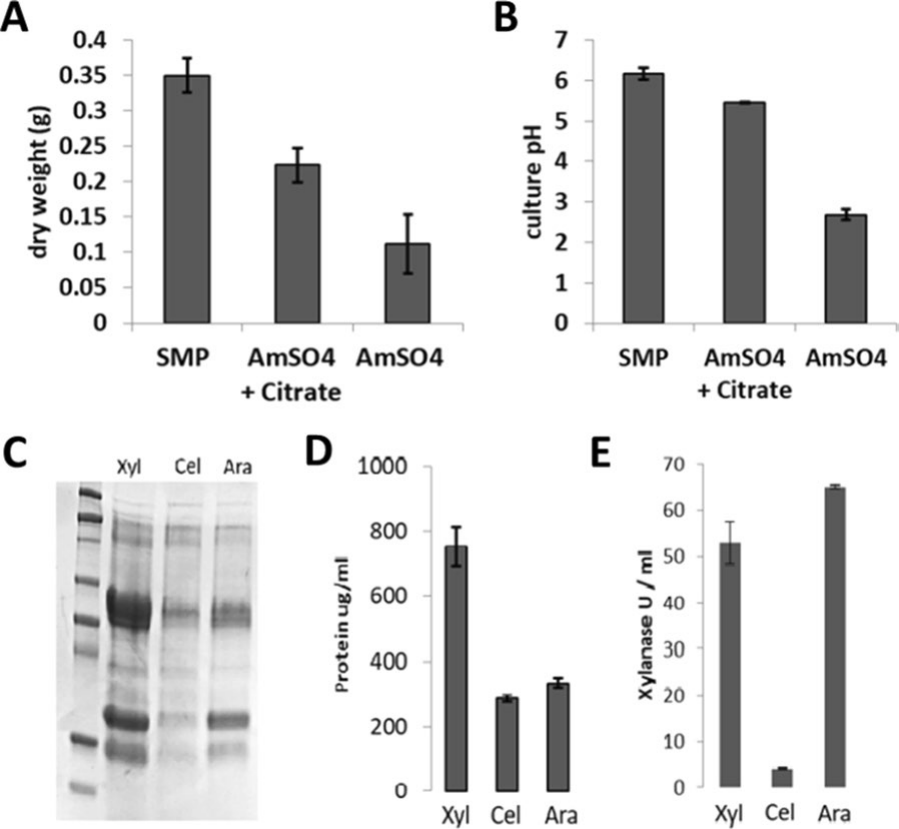
Minimal medium development for *T. aurantiacus* for performing RNA-Seq under fed-batch conditions. **A**, **B** Fungal growth characteristics in McClendon’s salts supplemented with soy meal peptone (SMP) or ammonium sulfate (AmSO_4_). Enzyme production in the new growth medium (25 mM AmSO_4_ and 25 mM sodium citrate) was assessed through fed-batch feeding of d-xylose, cellobiose and l-arabinose and tested with **C** SDS-PAGE (20 μl filtered supernatant broth loaded per lane), **D** Bradford Protein Assay and **E** DNS-xylanase assay (error bars represent standard deviations, *n* = 3)

*T. aurantiacus* growth on d-glucose with McClendon’s medium with soy meal peptone as the nitrogen source was compared with growth with (NH_4_)_2_SO_4_ (Fig. 2A and B). Growth of *T. aurantiacus* with (NH_4_)_2_SO_4_ as the nitrogen source in liquid medium was < 30% of the growth with soy meal peptone as measured by mycelial biomass. Growth with (NH_4_)_2_SO_4_ resulted in a drop in pH after 3 days from the initial of pH 5.5–2.5, while growth on soy meal peptone after 3 days resulted in pH of 6. The poor growth on (NH_4_)_2_SO_4_ was attributed to the drop in pH, which is consistent with previous studies demonstrating optimal growth and enzyme production by *T. aurantiacus* at pH ≥ 5 (12). To prevent the drop in pH during cultivation, the (NH_4_)_2_SO_4_-containing medium was buffered with 25 mM sodium citrate. The citrate buffer maintained the pH around five for the submerged *T. aurantiacus* culture and doubled the fungal growth compared to adding (NH_4_)_2_SO_4_ alone. This minimal medium was tested in a fed-batch experiment with d-xylose, cellobiose and l-arabinose as described before (Fig. 2). Although the overall protein production was lower in the minimal medium than in medium amended with soy meal peptone, the enzyme activity patterns were consistent between both tests. Therefore, this improved minimal medium was used to assess gene expression patterns during induction.

### Differential gene expression analysis of *T. aurantiacus*

The gene expression study was performed using the fed-batch system described above. *T. aurantiacus* was grown in d-glucose minimal medium and then shifted to shake flasks containing minimal medium, where d-xylose, cellobiose and l-arabinose were added through continuous feeding. Additionally, a starvation condition (only minimal medium without a carbon source) and a CAZyme-repressing condition (high d-glucose minimal medium) were added as controls.

**Fig. 3:**
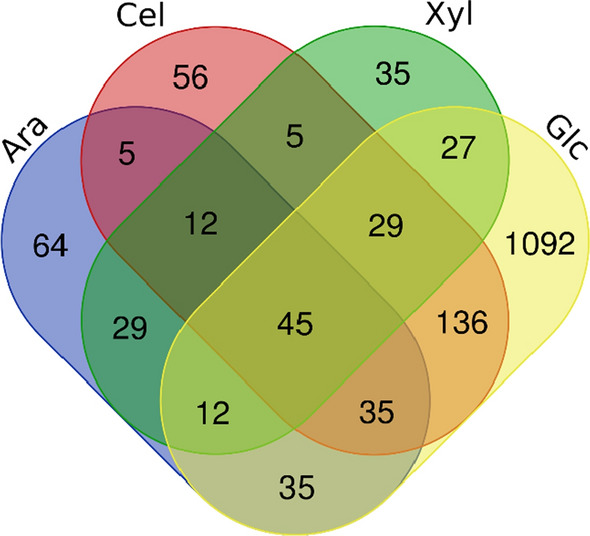
Venn diagram of differentially expressed genes that were up-regulated under arabinose feed (Ara), cellobiose feed (Cel), xylose feed (Xyl) and high glucose medium (Glc) compared to no carbohydrate medium. The figures represent the number of genes shared among the conditions as indicated

A Venn diagram was generated to uncover genes differently expressed in d-glucose medium and the sugar fed-batch conditions compared to no carbohydrate medium to investigate the effects of those sugars on gene expression (Fig. 3). All up-regulated genes of the d-xylose, cellobiose, l-arabinose and d-glucose treatments compared to no carbohydrate medium (logFC > 1, pval < 0.05) were used for this analysis. All numbers in parenthesis are *T. aurantiacus* protein IDs from the JGI Mycocosm database unless otherwise stated.

Genes specific for the l-arabinose fed-batch condition were a putative xylulokinase (Prot. ID: 63574) an arabinofuranosidase (Prot. ID: 51505) and an α-xylosidase (Prot. ID: 58732) together with a sugar transporter (Prot. ID: 44260) that is most closely related to the d-xylose transporter *xtrD* (AN0250) of *A. nidulans* [32].

For the d-xylose feed condition, no genes specific for xylan and C5 sugar metabolism were found except for a putative α-glucuronidase (Prot. ID: 36875) and an F-box protein orthologue (Prot. ID: 43769) of the *A. nidulans* gene *fbxA*. Deletion of *fbxA* caused reduced secretion of xylanases in its native host and impaired CCR, which was indicated by resistance of the *fbxA* mutant to the d-glucose analog 2-deoxy-d-glucose [33]. Additionally, an unknown sugar transporter (Prot. ID: 55223) was up-regulated. A putative transcriptional regulator with highest similarity to the *A. nidulans* sexual development regulator *nsdD* was found here, which was recently found to be vital for cellulase and xylanase in *P. oxalicum* [34].

When the genes specific for both C5 sugar feeds were investigated, we found two of the most highly secreted enzymes of *T. aurantiacus*: the xylanase (Prot. ID: 1236) and endoglucanase (Prot. ID: 65156) which have been identified earlier by proteomics [4]. Also, a putative β-xylosidase (Prot. ID: 64461), xylitol dehydrogenase (Prot. ID: 66263) and d-xylose dehydrogenase (Prot. ID: 63693) were identified, indicating expression of a variety of genes related to xylan hydrolysis and the d-xylose and l-arabinose catabolism of *T. aurantiacus*. The metabolism of those two C5 sugars has been extensively investigated for the related fungus *A. niger* [35]. Orthologous of all necessary enzymes for d-xylose and l-arabinose catabolism were identified in *T. aurantiacus*, except for the l-arabinose reductase, the first enzyme needed for l-arabinose assimilation (Additional file 1: Figure S1). All the genes in the *T. aurantiacus* d-xylose/l-arabinose assimilation pathway were highly up-regulated during l-arabinose feed, while d-xylose caused up-regulation of the same genes except the putative l-xylulose reductase orthologue. Another pathway that appeared to be up-regulated during the C5 sugar feeds was the unfolded protein response (UPR) (Additional file 1: Figure S2). Several components of this pathway, namely a *bipA* [36] orthologue (Prot. ID: 46710) was up-regulated in the l-arabinose condition and *clxA* [37] (Prot. ID: 65748) was highly expressed in the l-arabinose and d-xylose condition. We found that other UPR-related genes, such as the orthologue of the regulator that activates UPR, *hacA* [38] (Prot. ID: 7916), and the protein disulfide-isomerase *pdiA* [39] (Prot. ID: 64656), were highly expressed during d-xylose and l-arabinose feeding relative to all other conditions (Additional file 1: Figure S2). UPR genes are often expressed during fungal enzyme secretion to counteract protein folding stress [40].

Intriguingly, the cellobiose feed condition did not display any plant cell wall degradation related CAZymes that were specific for this condition. However, genes up-regulated during d-xylose, cellobiose and l-arabinose feed were the main secreted cellobiohydrolase (Prot. ID: 41785) and a β-glucosidase (Prot. ID: 38776). Therefore, the expression of the main secreted xylanase, endoglucanase and cellobiohydrolase together with several xylan degrading enzymes happened in the C5 sugar conditions, while the fungus in the cellobiose condition appeared to only express the cellobiohydrolase at high levels. Lastly, a serine carboxypeptidase (Prot. ID: 57314) was identified up-regulated in all sugar feeds, which had highest sequence similarity to *protH* of *A. niger*. This enzyme is predicted to be secreted, which could be involved in degradation of secreted CAZymes.

### Expression analysis of glycoside hydrolases, auxiliary family enzymes and carbohydrate esterases

The analysis of expression patterns was extended to compare expression of related CAZyme proteins. The carbohydrate active enzymes (CAZy) database classifies enzymes relevant for the degradation, modification or creation of glycosidic bonds [41]. The JGI mycocosm protein portal contains those CAZyme annotations for *T. aurantiacus*, where 323 CAZy genes are annotated in the *T. aurantiacus* genome [42]. Enzymes belonging to the glycoside hydrolases (GH), auxiliary activity (AA) and carbohydrate esterases (CE) have been found to be most important for plant cell wall deconstruction [14]. Five CAZymes (Prot. ID: 3070, AA 9; 41785, GH7; 65156, GH5; 1236, GH10; 46699, GH3) have been previously identified in *T. aurantiacus* ATCC 26904 from liquid cultures grown on plant biomass by proteomics analysis [3]. Virtually nothing is known about what other genes of this fungus respond to plant polysaccharides and their breakdown products. To uncover the expression trends of those unknown genes, we generated a heat map of the expression trends of the *T. aurantiacus* GH, AA and CE families.

**Fig. 4:**
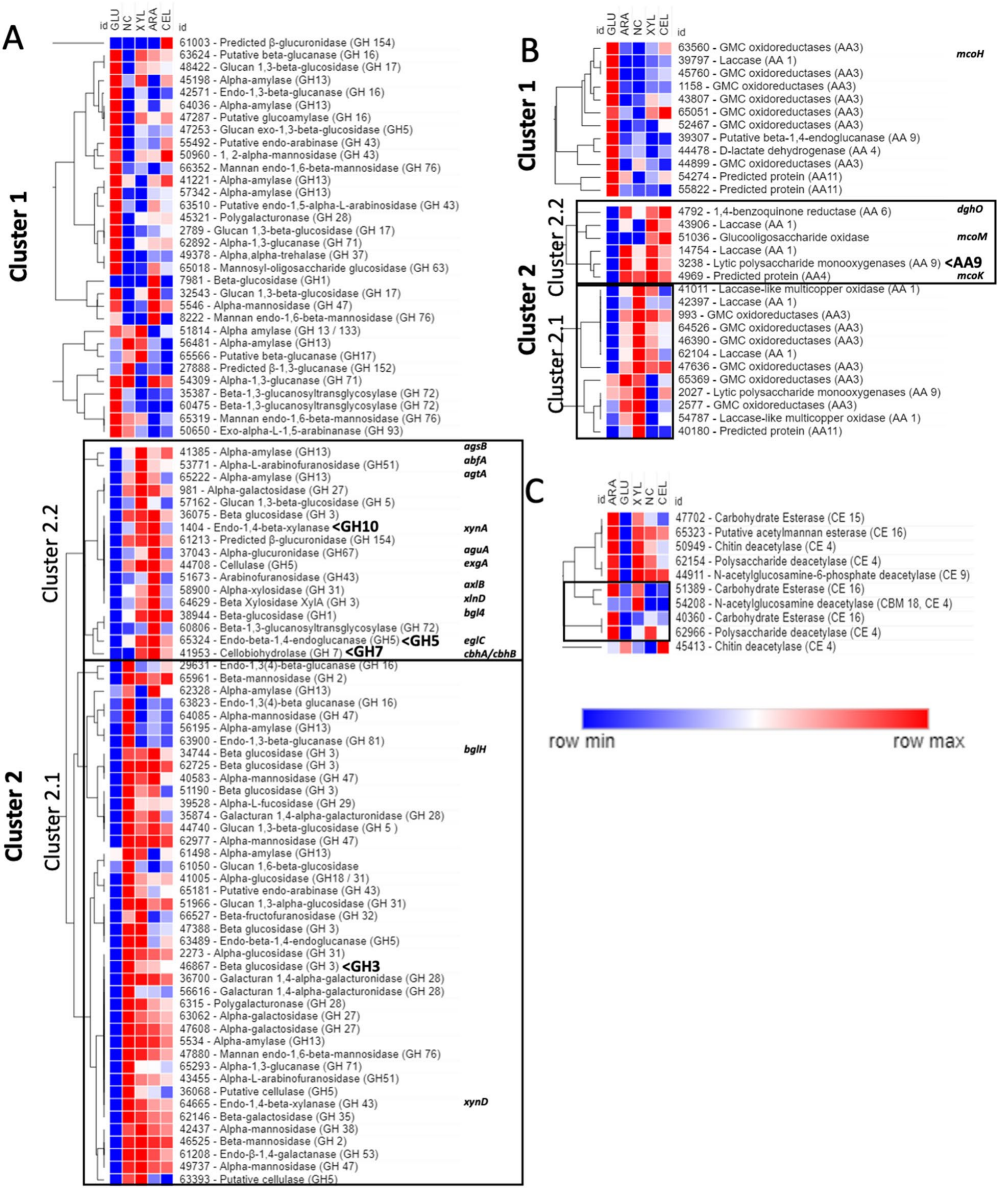
Gene expression analysis of **A** glycoside hydrolases, **B** oxidoreductases and **C** esterases related to plant polysaccharide deconstruction and modification. Each colored cell represents the average of three biological replicates. The known CAZymes of *T. aurantiacus* that have been shown to be secreted during growth and cellulosic plant biomass that have been verified using shotgun proteomics are indicated (GH3-10 and AA9) [4]. Additionally the gene names of the closest matches to known *A. niger* CAZymes determined via BLAST are indicated in italic letters

The GH heat map (Fig. 2a) contained two main clusters: cluster 1 showed GHs that were highly up-regulated on d-glucose or d-glucose and other conditions, while cluster 2 contained GHs that exhibited low expression on d-glucose. Cluster 1 contained diverse types of GHs with only few predicted types of GH related to cellulose, xylan and pectin deconstruction. Conversely, the main four secreted GH described above were all found in cluster 2 (Fig. 4a). Cluster 2 also contained most of the cellulase, xylanase and pectinase genes and was divided into two sub-clusters (Cluster 2.1 and 2.2). Cluster 2.1 contained GHs that were highly up-regulated in no carbohydrate medium and to different degrees up-regulated during the sugar feed conditions. Thus, cluster 2.1 comprised GH that most likely resemble starvation responsive genes. The β-glucosidase that was previously identified in the *T. aurantiacus* supernatant [3]was also found to be strongly expressed during starvation and to a lesser extend during the sugar feed conditions. However, Cluster 2.2 contained GHs that were highly expressed under the sugar feed conditions and showed lower expression during starvation. This cluster appeared to resemble the genes induced by the sugar feed conditions and contains the previously identified endoglucanase, cellobiohydrolase and xylanase (marked). Also, most of cluster 2.2 genes are putative orthologues of *A. niger* GHs linked to plant cell wall deconstruction (Fig. 4a). *Aspergillus* *niger* orthologues of the GH51 arabinofuranosidase *afbA*, was found to released l-arabinose from arabinan and an unknown putative GH5 (T.ID: 44708) with highest similarity to *exgC* in *A. nidulans* were found highly expressed on d-xylose. Genes highly expressed on l-arabinose showed high similarity to the α-glucuronidase *aguA*, α-xylosidase *axlB* and the β-xylosidase *xlnD*. In addition to the high expression of the main secreted GH5, 7 and 10, we also found another putative GH5 that had highest similarity to *exgA* and was highly expressed on both C5 sugars. A putative β-glucosidase with highest similarity to *bgl4* was the only GH highly expressed under all three inducing conditions. Few GHs showed elevated expression on cellobiose with only one gene exclusively expressed under this condition predicted β-glucuronidase (Prot. ID: 61003) that had the highest similarity to two uncharacterized *A. nidulans* genes: AN7089 and AN3992. Genes highly expressed during cellobiose feed in cluster 2.2 were mostly cellulases (GH1, GH3, GH5 and GH7). This is in accordance with our finding that cellobiose feed led to only weak xylanase activity in the culture supernatant while activity was high for glucose release from Avicel (Fig. 1).

Similar trends were found for the AA heat map (Fig. 4b), where cluster 1 contained AAs that were highly expressed on d-glucose with almost no expression on all other conditions. Cluster 2 was divided in two sub-clusters. Cluster 2.1 contained AA that were highly expressed during starvation including four predicted laccases, whose expression is often associated with starvation also in other fungi, and one putative unknown LPMO (Prot. ID: 2027). Cluster 2.2 contained genes that displayed low expression on d-glucose and no carbohydrate medium, and high expression during the sugar feed conditions. Here, the well-studied *T. aurantiacus* LPMO was found highly expressed during d-xylose and l-arabinose and to a less extent on cellobiose. Two predicted laccases showed a similar trend. Interestingly, one further laccase (Prot. ID: 39797) was highly expressed on d-glucose. This gene has a high similarity to *A. niger mcoH* and *Trichophyton benhamiae* conidial pigment biosynthesis oxidase. Therefore, this laccase might be required for pigmentation and could serve as a color locus for strain engineering efforts to identify targeted integrations of DNA constructs, using the newly developed transformation procedure for *T. aurantiacus* [6]*.*

The CE expression heat map (Fig. 4c) revealed that all genes displayed virtually no expression on d-glucose, with the exception of a putative chitin-deacetylase (Prot. ID: 45413). Most CEs showed high expression on both d-xylose and l-arabinose and low expression on cellobiose, consistent with their deconstruction of bonds in hemicellulose. None of these genes had high similarities to genes characterized in *Aspergilli*. The CEs that might likely be of interest for lignocellulose deconstruction are those four that showed low expression on d-glucose and no carbon and thus seemed to be specifically expressed on one or both C5 sugars (Fig. 4c, black frame).

## Discussion

The understanding of fungal CAZyme induction is fundamental to enable the efficient conversion of lignocellulose into biofuels and bio-based products. In this study, we compared the CAZyme-inducing effect of d-xylose, cellobiose and l-arabinose through a fed-batch system at shake-flask scale using a peristaltic pump. Here, it was found that continuously feeding d-xylose, cellobiose and l-arabinose caused elevated secretion of cellulases of *T. aurantiacus*, which was not observed when the same amount of sugar was added in the beginning of the experiment. Notably, we found that while stimulating high enzyme secretion, individual enzymatic activities depended on which sugar was fed to the medium. Xylanase activity was almost absent in the cellobiose feed cultures, while this activity was highly induced in the C5 sugar feed cultures. While overall CMCase activity was similar among all three sugar feeds, we found elevated cellobiosidase activity during C5 sugar feeding and elevated β-glucosidase activity during cellobiose feeding. Cellobiose feeding also resulted in culture supernatants with an increased ability to release sugars from Avicel. The reason for lower enzyme secretion during batch cultivations may be that simple sugars can cause CCR when present at high amounts, which is partly mediated through the carbon catabolite repressor Cre-1/Cre1/CreA (22, 23, 45, 46)[21, 22, 43, 44]. Typically, secretion of CAZyme for the breakdown of complex sugars, such as polysaccharides, is repressed until all simple sugars are reduced to low levels. However, once the sugars are depleted, there will be no further induction in batch media. Complex lignocellulosic substrates, however, are broken down over longer periods of time and the sugar release thus happens more slowly and continuously, similar to a fed-batch process. Fed-batch has been found to improve many aspects of enzyme production in bioreactors with filamentous fungi compared to batch cultivations, indicating the importance of regulated substrate levels [20, 45]. We argue that fed-batch tests are vital for understanding the potential of individual inducer molecules, which is not feasible when using complex carbon sources such as cellulose or xylanase.

The high level of CAZyme production when feeding purified sugars illuminates previous studies of fungal gene expression and enzymatic activity, which reported that simple sugars such as d-xylose, cellobiose and l-arabinose during batch fermentations weakly induced cellulases and xylanases compared to complex polysaccharides such as xylan and lignocellulosic plant biomass in *T. aurantiacus* [9], *A. niger* [15] and *Talaromyces versatilis* [46]*.* These studies might have found stronger induction effects of the simple sugars on cellulases and xylanases expression when applying continuous feeding as we observed for *T. aurantiacus*. Nevertheless, it is widely established that complex substrates induce a broader variety of CAZymes [14]. Feeding mixtures of sugars or hydrolysates may generate more efficacious enzyme formulations without adding solid substrates to the fermentation medium [46–48].

The gene expression study established that cellulase and xylanase induction in *T. aurantiacus* follows a pattern similar to the related fungus *A. niger*, where d-xylose was found to induce production of these enzymes [15]. Putative *T. aurantiacus* orthologues of most of the characterized cell wall degrading enzymes from *A. niger* were highly up-regulated during d-xylose and l-arabinose feeding. These highly expressed *T. aurantiacus* genes have high similarity to *A. niger* orthologues related to deconstruction of cellulose (*exgA*, *eglC*, *cbhA*/*cbhB* and *bgl4*), xylan (*xynA*, *axlB* and *xlnD*) and arabinan (*abfA*). Notably, for the cellobiose feed, expression of all the respective *T. aurantiacus* genes was substantially lower compared to the C5 sugar feeds, with only the putative *bgl4* orthologue showing equally high expression. Only putative orthologues of *exgA*, *bgl4*, *eglC* and *cbhA*/*cbhB* showed increased expression during cellobiose feed. These observations indicated that cellobiose mainly induced cellulase genes. We also found that the prominent *T. aurantiacus* LPMO [5] showed highest expression during C5 sugar feeds and intermediate expression during cellobiose feeding.

The effect of d-xylose feeding was different for the *T. reesei* CL847 mutant where this sugar strongly induced xylanase activity but very little cellulase activities while cellobiose and lactose strongly induced cellulase activities [20]. Furthermore, feeding a mixture of d-xylose and cellobiose or lactose led to reduction in cellulase activities compared to not feeding d-xylose. In contrast, feeding a mixture of d-xylose, d-glucose and cellobiose did not decrease cellulase production of the *T. reesei* M3-1 mutant compared to not adding d-xylose to the feed, while xylanase secretion was substantially promoted by adding d-xylose [17]. This contrary observation could be due to the different mutant strains used in the respective studies. However, unlike industrial hosts such as *Trichoderma *sp., d-xylose and l-arabinose alone can be used to produce cellulases and xylanases simultaneously at high amounts in *T. aurantiacus*.

The genetic regulation of *T. aurantiacus* CAZyme production at this point remains unknown. In *A. niger* and *T. reesei* it was found that both types of enzymes were induced by the same orthologues (XlnR/Xyr1) [14, 15]. Notably, XlnR in *A. niger* was found to induce expression of *xlnD* and *aguA* when grown on d-xylose and xylan [15]. Both putative CAZyme orthologues were also highly expressed in *T. aurantiacus* when grown on d-xylose. This finding, together with the recent demonstration that XlnR overexpression in *T. aurantiacus* caused a substantial increase in xlyanase production compared to the wild type strain [6], suggests that XlnR has a similar role in *T. aurantiacus* as in *A. niger*. Additonally, two further orthologues (Prot. IDs 43769 and 600557) of the f-box protein gene *fbxA* of *A. nidulans* and the sexual development regulator *nsdD* were identified through Venn diagram binning in the xylose condition in *T. aurantiacus*; *fbxA* was found to be essential for xylanase expression in *A. nidulans* [33]. Recently, the *nsdD* orthologue was deleted in *P. oxalicum*, which caused a substantial reduction of cellulase and xylanase secretion [34]. Thus, 600557 might have a similar function in *T. auranticus* and could be another target for strain improvement. Further transcription factors that have been found to mediate CAZyme production were orthologues of carbon catabolite repressor Cre1/Cre-1/CreA [43, 44, 49], the cellulase/xylanase regulators XlnR/Xyr1, Clr-1/ClrA, Clr2/ClrB and ClbR [14, 15, 48, 50], the amylase regulator AmyR/Col26 [51–53], the nitrogen- and starvation regualtor PacG/Vib1[52] and further regulators involved in pectin and rhamnose utilization GaaR/Pdr-1/RhaR [54–56]. Putative orthologues for all of those genes were found in *T. aurantiacus* and their dynamics could be uncovered using our data set. We found that the putative *T. aurantiacus* orthologues of all of the genes listed above except ClbR and CreA showed statistically significant lower expression on d-glucose (pval < 0.05) compared to most other feed and starvation conditions (Additional file 1: Figure S3). The comparably high expression of those regulators on the no carbohydrate medium compared to the sugar feed conditions is not surprising, since some transcription factors such as CRE1 and XLNR were found to be regulated at the protein level through post-translational modifications in other fungi [57–62]. Nevertheless, CCR seemed to affect those regulators in a similar way, potentially mediated by the CreA orthologue, as it has been described for *A. niger*, *N. crassa* and *T. reesei*. Therefore, we hypothesize that overexpression of the respective *T. aurantiacus* genes showing high similarity to known transcriptional CAZyme activators will likely have substantial effects on expression of cellulases, xylanases and pectinases. Furthermore, we found that several gene orthologues related to UPR in *A. niger* were found to be highly expressed during d-xylose and l-arabinose feed in *T. aurantiacus*. The observation of upregulation of genes associated with UPR is consistent with previous observations in *A. niger* of stress responses under conditions where high levels of protein are secreted [60, 61]. In *N. crassa*, disruption of UPR-related genes did not effect the transcription of CAZyme genes when grown on cellulose, but disrupted the secretion of their gene products [62]. While it is not certain at this point whether this pathway is stimulated by C5 sugars or the onset of high enzyme secretion induced by those sugars, the UPR pathway may be a promising target for future strain engineering efforts. Lastly, we identified several candidate genes in *T. aurantiacus* that had highest similarity to genes used by *A. niger* to assimilate d-xylose and l-arabinose. While experimental studies of their activities are required to draw final conclusions, their high up-regulation in *T. aurantiacus* during growth on l-arabinose and d-xylose makes them the suitable targets for studying C5 sugar catabolism in this fungus.

## Conclusion

This study has identified three lignocellulose-derived sugars, d-xylose, l-arabinose and cellobiose, as CAZyme inducers for *T. aurantiacus*. Gene expression analysis indicates that the C5 sugars induce the production of a broad spectrum of CAZymes, including cellulases and xylanases while cellobiose primarily induces cellulases. The observation of broad CAZyme induction by C5 sugars is similar to what has previously observed for CAZyme induction in *A. niger*.

## Methods

### Chemicals

All chemicals were purchased from MilliporeSigma (St. Louis, MO, USA) unless otherwise noted.

### Strains and culture conditions

*Thermoascus aurantiacus* ATCC 26904, which was obtained from the American Type Cell Culture Collection and maintained on potato dextrose agar by inoculating fresh plates with a piece of a grown culture. Cultures were incubated for 2 days at 50 °C to enhance growth and were then shifted to 45 °C to prevent desiccation of the plates. All incubators (Complete Culture Control Incubator CCC 2.5d, Boekel Scientific, Feasterville-Trevose, PA, USA) contained several water reservoirs to keep the air moist and prevent plate desiccation. New fungal cultures were periodically inoculated from cryostocks.

### Induction experiments

From a 6-day-old agar plate culture, *T. aurantiacus* ascospores were harvested by pouring 5 ml of autoclaved water on the plate surface and gently scraping the plate with disposable cell spreaders (Heathrow Scientific, Vernon Hills, IL, USA). The spores were transferred to a sterile 15 ml Falcon tube (Cellstar^®^, Greiner Bio-One, Monroe, NC, USA) and continuously vortexed and were then used as an inoculum (10^6^ spores/ml) for 300 ml glucose medium pre-cultures [McClendon’s salts (4), 2% glucose (w/v) and 0.8% soy meal peptone]. The pre-cultures were incubated for 48 h at 50 °C in a rotary shaker (Multitron, American Laboratory Trading, San Francisco, CA, USA). After 48 h, all pre-cultures were combined and filtered in vacuo under sterile conditions over a glass fiber filter-based system until the mycelia were dry. The mycelia (1.5 g) were weighed into baffled 250-ml Erlenmeyer culture flasks containing 50 ml of culture medium that did not have a carbon substrate. The carbon sources (d-xylose, cellobiose, l-arabinose) were autoclaved separately and added to 1% w/v for the batch cultures. The batch cultures were sealed with foam stoppers and incubated for 72 h at 50 °C at 180 rpm. For the fed-batch induction experiments, sterile solutions of the sugar were added by a peristaltic pump [BT100-1L Multi-Channel Peristaltic Pump (Langer Instruments Corp., Boonton, NJ, USA)] according to a previously established protocol [11].

### Thermoascus minimal medium development

*Thermoascus aurantiacus* cultures for minimal medium development were performed by comparing growth with 50 ml of modified McClendon’s medium (McClendon’s salts, 2% glucose and 0.8% soymeal peptone) to a medium where the peptone has been replaced by 25 mM (NH_4_)_2_SO_4_ or 25 mM (NH_4_)_2_SO_4_ and 25 mM trisodium citrate. The cultures were inoculated with 10^6^ spores in 250-ml baffled Erlenmeyer flasks, and incubated on a rotary shaker at 50 °C and 180 rpm. The peptone cultures were incubated for 48 h and the (NH_4_)2SO4 cultures were incubated for 72 h. The pH determination was performed with a calibrated pH meter (SYMPHONY SB70P, VWR, Radnor, PA, USA) and the dry weight of the mycelia was measured by filtering the content of an entire culture over a previously weighed filter paper with a vacuum system and washing it several times with ddH_2_O. The mycelia were dried for 48 h at 65 °C and weighed. Induction of *T. aurantiacus* cellulases and xylanases was performed by fed-batch induction with the peristaltic pump as described above with the culture medium containing 25 mM (NH_4_)_2_SO_4_ and 30 mM trisodium citrate instead of 0.8% soy meal peptone.

### Cultivations for gene expression analysis

For gene expression analysis, *T. aurantiacus* pre-cultures were grown as described above with the minimal medium containing 25 mM (NH_4_)_2_SO_4_ and 30 mM trisodium citrate and the mycelia from these pre-cultures isolated and inoculated into fed-batch shift experiments as described above, The peristaltic pump was calibrated to release 50 mg/l/h of either d-xylose, l-arabinose or cellobiose during 16 h to four-shake flask replicates for each sugar. In addition to these cultures, four cultures were set up as a no carbohydrate control and four cultures containing 2% (w/v) d-glucose served as a control treatment for carbon catabolite repressing conditions. After 16 h, the feed was stopped and 10 ml of each culture was rapidly filtered over a filter paper, peeled off and snap-frozen with liquid nitrogen. These frozen mycelia were ground with a mortar and a pestle and stored at − 80 °C. The RNA extraction was performed with Maxwell RSC system (Promega, Madison, WI, USA) and the Plant RNA kit (Promega, Madison, WI, USA) according to the fungal RNA-extraction protocol. Briefly, aliquots of extracted mycelium were transferred into designated tubes and 400 μl of homogenization solution and 200 μl of Lysis buffer were added and vortexed and the extraction was initiated. The purified RNA was eluted in nuclease-free water. RNA quality was assessed with a NanoDrop spectrometer and the quality with the RNA 6000 Nano kit (Agilent, Waldbronn, Baden-Württemberg, Germany). RNA concentration was determined with the Qubit RNA HS Assay Kit (ThermoFisher Scientific, Waltham, MA, USA) and sent to the UC Davis DNA Technologies Core for sequencing. With these RNA samples, the library preparation, sequencing and the preliminary differential gene expression calculations were performed by UC Davis DNA Technologies Core through the 3′Tag-Seq method (https://dnatech.genomecenter.ucdavis.edu/tag-seq-gene-expression-profiling/) (https://doi.org/10.1186/s12864-018-5393-3). The libraries were sequenced using a HiSeq 4000 instrument (Illumina).

### Gene expression analysis

The Tag-Seq reads were aligned to the *T. aurantiacus* genome (https://genome.jgi.doe.gov/portal/pages/dynamicOrganismDownload.jsf?organism=Theau1) with STAR v2.5.5.2b [63] to generate a table containing the counts per gene. Furthermore, 500 bases were added to the coordinates of the 3′ end of each transcript (or until the start of the next gene) to improve annotation (from only 21% of the reads aligned, to 77%). Genes with mean expression levels lower than one were filtered which resulted in a table of 8591 genes (instead of the original 8798 genes). This minimal filtering step ensured that genes with low expression such as transcriptional regulators remained in the data set. The differential expression analysis was performed with the DESeq2 package in R [64]. Heat maps were created using [pheatmap(sampleDistMatrix)] with the pheatmap package and the Morpheus online tool (https://software.broadinstitute.org/morpheus/). For generating the Venn diagram, the genes were grouped in several Venn bins using the software supplied at: http://bioinformatics.psb.ugent.be/webtools/Venn/. For the heatmaps, the list of annotated CAZyme genes in *T. aurantiacus* was matched with the corresponding filtered expression values for each condition (see Additional file 2). To obtain the Z-score for each condition, the mean calculated for each condition was subtracted from each value and then divided by the standard deviation. Hierarchical clustering with the Spearman’s rank correlation as a metric was used to cluster the rows (genes) and columns (conditions).

### Protein analysis

Culture filtrates for protein analysis were recovered by transfer of the culture broth to a spin column, which was centrifuged at 8000 rpm for 3 min. The filtrate from the column was then used for protein assays.

The protein profile was investigated with SDS-PAGE gels (8–16% Tris–glycine mini gel; Invitrogen, Carlsbad, CA, USA). Accordingly, 15 μl of the filtered culture broth was mixed in a 5:1 ratio with loading dye [Laemmli buffer/2-mercaptoethanol (4:1)] and boiled for 5 min at 95 °C. The mixture was loaded together with 5 μl of the Novex sharp prestained protein standard molecular weight markers (Thermo Fisher Scientific, Waltham, MA USA) and separated for 65 min at 140 V. The gel was then stained with SimplyBlue safe stain (Thermo Fisher Scientific, Waltham, MA USA).

Protein concentrations were measured with the Bradford Protein Assay (Bio-Rad, Hercules, CA, USA). The assay reagent was diluted 1:5 and 200 ml were added to a 96-well plate together with 5 μl of culture filtrate. Bovine gamma globulin (Thermo Fisher Scientific, Waltham, MA USA) was added as a standard (0–2 g/l) [11]. The absorbance measurement was performed at 595 nm.

CMCase and xylanase activity was measured with the DNS method [65] with 1% (w/v) sodium carboxymethylcellulose (CMC) or beechwood xylan as substrate in 50 mM sodium acetate buffer pH 5. For the assay, culture filtrates were diluted either 1:20 or to 50 μg protein/ml and 10 μl were added in triplicates to a 96-well PCR plate together with 70 μl of the polysaccharide substrates. Monomeric sugar standards (d-glucose for CMCase and d-xylose for xylanase) ranging from 0 to 12.5 uM was added in duplicates to each plate. All assays were performed in PCR cyclers for 30 min at 65 °C. The reactions were stopped via adding 80 μl of the DNS reagent and boiling for 5 min at 95 °C. The amount of released sugars was measured at *λ* = 540 nm. One unit of enzyme activity (U/ml) was defined as amount of released sugar (nmol) per time (min) per volume of culture supernatant (ml). Assays for *p*-nitrophenyl β-d-cellobioside (pNPC) and *p*-nitrophenyl β-d-glucopyranoside (pNPG) were performed as previously described [66].

Glucose release from Avicel (2%) was measured by diluting filtrates (40 μl) from d-xylose (0.90 mg/ml), l-arabinose (0.61 mg/ml) and cellobiose (0.56 mg/ml) cultures into 200 μl of 50 mM NaOAc buffer pH 5.0 containing 0.6 mM ascorbate and incubating the reactions at 50 °C and 180 rpm for 24 h. After 24 h, glucose was measured using a YSI 2500 (YSI Incorporated, Yellow Springs, Ohio, USA).

## Supplementary Information

**Additional file 1: Figure S1.** Overview of the putative D-xylose and L-arabinose assimilation pathway in *T. aurantiacus*. The table contains a heat map of the putative pathway genes shown below. The predicted orthologues of *A. niger* are shown on the right hand side. Enzymes and intermediates of the pathway are shown below, where high expression is indicated during L-arabinose (Ara) or D-xylose (Xyl) feed or NA if the predicted gene could not be identified for *T. aurantiacus*. Red indicates high and blue low gene expression. **Figure S2.** Comparison of genes related to the unfolded protein response in *T. aurantiacus* under different growth conditions. Bars (mean) and error bars (standard deviation) were calculated from 3 biological replicates, asterisks indicate statistical significance compared to the no carbohydrate condition (pval < 0.05). **Figure S3.** Regulation trends of *T. aurantaicus* genes that are putative orthologues of transcriptional CAZy regulators in *A. niger*. Numbers represent *T. aurantaicus* protein IDs from the JGI MycoCosm database (43) and gene names refer to the *A. niger* genes showing the highest similarity based on BLAST searches using gene sequences. Conditions are indicated as follows: Xyl, Cel and Ara = D-xylose, cellobiose and L-arabinose feed, Glu = high D-glucose medium and NC = no carbohydrate medium. Each bar represents the average of 3 biological replicates and error bars are standard deviations of those replicates. A statistical significant difference to the glucose codnitons is marked with asterisks below each bar (pval < 0.05).

**Additional file 2.** Tables for differential expression by* T. aurantiacus* induced with C5 and C6 sugars. Data is shown for genes whose differential expression has an adjusted pval < 1.

## Data Availability

The RNA-seq datasets generated and/or analyzed during the current study are available in the NCBI Sequence Read Archive (SRA) in Bioproject PRJNA681608 under Biosamples SAMN19237569–SAMN19237583.
