# Supplementary material for: CAZymes from the thermophilic fungus Thermoascus aurantiacus are induced by C5 and C6 sugars
Source: Biotechnol Biofuels. 2021 Aug 12;14:169. doi: 10.1186/s13068-021-02018-5 (PMC8359064; doi:10.1186/s13068-021-02018-5)
Supplement: Supplementary file 1 — Additional file 1: Figure S1. Overview of the putative D-xylose and L-arabinose assimilation pathway in T. aurantiacus. The table contains a heat map of the putative pathway genes shown below. The predicted orthologues of A. niger are shown on the right hand side. Enzymes and intermediates of the pathway are shown below, where high expression is indicated during L-arabinose (Ara) or D-xylose (Xyl) feed or NA if the predicted gene could not be identified for T. aurantiacus. Red indicates high and blue low gene expression. Figure S2. Comparison of genes related to the unfolded protein response in T. aurantiacus under different growth conditions. Bars (mean) and error bars (standard deviation) were calculated from 3 biological replicates, asterisks indicate statistical significance compared to the no carbohydrate condition (pval < 0.05). Figure S3. Regulation trends of T. aurantaicus genes that are putative orthologues of transcriptional CAZy regulators in A. niger. Numbers represent T. aurantaicus protein IDs from the JGI MycoCosm database (43) and gene names refer to the A. niger genes showing the highest similarity based on BLAST searches using gene sequences. Conditions are indicated as follows: Xyl, Cel and Ara = D-xylose, cellobiose and L-arabinose feed, Glu = high D-glucose medium and NC = no carbohydrate medium. Each bar represents the average of 3 biological replicates and error bars are standard deviations of those replicates. A statistical significant difference to the glucose codnitons is marked with asterisks below each bar (pval < 0.05). [file 13068_2021_2018_MOESM1_ESM.docx]

**CAZymes from the thermophilic fungus Thermoascus aurantiacus**

**are induced by C5 and C6 sugars**

**Raphael Gabriel^1,2,3^, Rebecca Mueller^1,2,3^, Lena Floerl^1,2,4,5^, Cynthia Hopson^1,2,6^, Simon Harth^1,2,7^, Timo Schuerg^1,2^, Andre Fleissner^3^ and Steven W. Singer^1,2*^**

Additional file 1: Figures 1S–S3


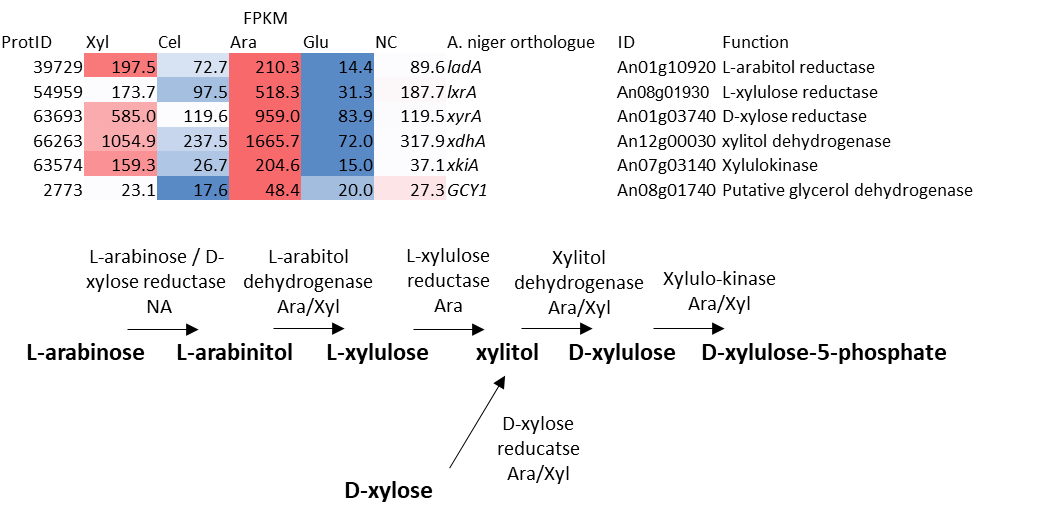


Additional file 1: Figure S1. Overview of the putative D-xylose and L-arabinose assimilation pathway in *T. aurantiacus*. The table contains a heat map of the putative pathway genes shown below. The predicted orthologues of *A. niger* are shown on the right hand side. Enzymes and intermediates of the pathway are shown below, where high expression is indicated during L-arabinose (Ara) or D-xylose (Xyl) feed or NA if the predicted gene could not be identified for *T. aurantiacus*. Red indicates high and blue low gene expression.

**
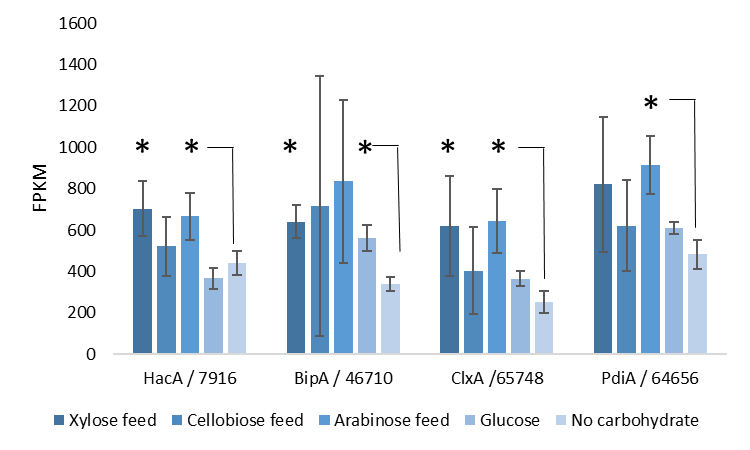
**

Additional file 1: Figure S2. Comparison of genes related to the unfolded protein response in *T. aurantiacus* under different growth conditions. Bars (mean) and error bars (standard deviation) were calculated from 3 biological replicates, asterisks indicate statistical significance compared to the no carbohydrate condition (pval < 0.05).

**
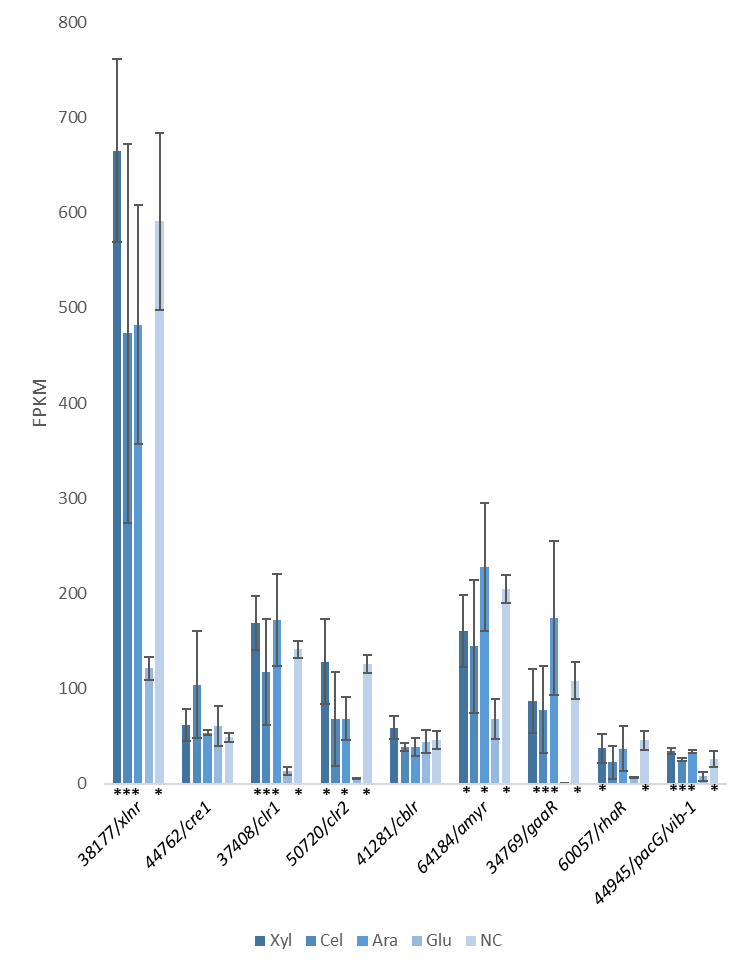
**

Additional file 1: Figure S3. Regulation trends of *T. aurantaicus* genes that are putative orthologues of transcriptional CAZy regulators in *A. niger*. Numbers represent *T. aurantaicus* protein IDs from the JGI MycoCosm database (43) and gene names refer to the *A. niger* genes showing the highest similarity based on BLAST searches using gene sequences. Conditions are indicated as follows: Xyl, Cel and Ara = D-xylose, cellobiose and L-arabinose feed, Glu = high D-glucose medium and NC = no carbohydrate medium. Each bar represents the average of 3 biological replicates and error bars are standard deviations of those replicates. A statistical significant difference to the glucose codnitons is marked with asterisks below each bar (pval <0.05).
